# Strategies for increasing gait speed in patients with hip osteoarthritis: their clinical significance and effects on hip loading

**DOI:** 10.1186/s13075-021-02514-x

**Published:** 2021-04-28

**Authors:** Hiroshige Tateuchi, Haruhiko Akiyama, Koji Goto, Kazutaka So, Yutaka Kuroda, Noriaki Ichihashi

**Affiliations:** 1 Department of Physical Therapy, Human Health Sciences, Graduate School of Medicine, Kyoto University, 53 Kawahara-cho, Shogoin, Sakyo-ku, Kyoto, 606-8507 Japan; 2 Department of Orthopaedic Surgery, School of Medicine, Gifu University, Gifu, Japan; 3 Department of Orthopaedic Surgery, Graduate School of Medicine, Kyoto University, Kyoto, Japan; 4 Department of Orthopaedic Surgery, Osaka Red Cross Hospital, Osaka, Japan

**Keywords:** Hip osteoarthritis, Gait, Physical function, Pain, Joint moment

## Abstract

**Background:**

Changes in gait speed are required in various situations and can be achieved by changing stride length, cadence, or both. Differences in strategies for increasing gait speed may have different effects on hip joint and physical function. The purpose of this study was to determine the effects of strategies for increasing gait speed on hip pain, physical function, and changes in hip loading during gait in patients with hip osteoarthritis (OA). We hypothesized that patients who increase gait speed mainly by increasing cadence would have lesser hip pain, a higher physical function, and a lower rate of increase in hip moments with increasing gait speed.

**Methods:**

Forty-seven patients with secondary hip OA (age, 48.3 ± 11.0 years) were included. Gait speed, stride length, cadence, and peak and impulse of the hip moments were measured during gait at self-selected normal and fast gait speeds. The patients were classified as types S (with mainly increasing stride length, *n* = 11 [23.4%]), C (with mainly increasing cadence, *n* = 23 [48.9%]), and SC (with increasing stride length and cadence, *n* = 13 [27.7%]) according to whether they used changes in stride length and/or cadence to transition from normal to fast gait. Hip pain, physical function, and hip moment changes during gait were compared between types.

**Results:**

The physical function was higher in types C (38.0 ± 8.8, *P* = 0.018) and SC (40.6 ± 8.5, *P* = 0.015) than in type S (28.2 ± 7.8), even after adjustment for age and minimum joint space width. Hip pain was not significantly different between types. The robustness of these results was confirmed with sensitivity analysis. The rates of increases in peak external hip adduction (*P* = 0.003) and internal rotation moments (*P* = 0.009) were lower in type C than in type SC.

**Conclusions:**

Type C tended to suppress the increase in hip moments during fast gait. Types C and SC, which included increased cadence, maintained higher physical function levels than type S. Encouraging the use of cadence-increasing strategy may be useful for reducing hip loading and maintaining physical function in patients with hip OA.

**Supplementary Information:**

The online version contains supplementary material available at 10.1186/s13075-021-02514-x.

## Background

Changes in gait speed are required in various situations in daily life, such as ambulating outdoors, including crosswalks [1]. As gait speed is determined by stride length and cadence, increased gait speed can be due to increased stride length, increased cadence, or a combination of both [2, 3]. In healthy individuals, both stride length and cadence increase linearly with increasing gait speed, regardless of age [2]. This stride length-cadence relationship represents the central control of automatic gait. Moreover, loading on the lower limb joints can increase as the gait speed increases [3–7]. Increasing stride length rather than cadence increases the peak external joint moment on hip flexion, hip adduction, hip internal rotation, knee flexion, knee adduction, and ankle plantarflexion [3, 4, 6, 7]. Therefore, differences in the strategies (i.e., increased stride length, increased cadence, or both) used to change gait speed could alter the loading on the lower limb joints. Given that the difference in the strategy affects joint loading, understanding the strategies associated with changes in gait speed is important for assisting patients with joint diseases.

A decrease in gait speed mainly by decreasing stride length is a typical characteristic of the spatiotemporal gait parameters in patients with hip osteoarthritis (OA) [8]. Although the stride length and cadence increase as the gait speed increases, for patients with hip OA as a whole, increases in stride length and cadence in these individuals tend to have a wider data distribution than in healthy individuals [9]. This may indicate variations in strategies to increase gait speed in patients with hip OA. However, little is known about these strategy variations.

Differences in the types of strategies for increasing gait speed may change hip joint loading, even with equal increases in gait speed; consequently, differences in strategies might affect hip joint symptoms and physical function status. However, the relationship between the type of strategy used to increase gait speed revealed by gait analysis at different speeds and hip pain and physical function levels has not been investigated in individuals with hip OA. Examining the strategies for changes in gait speed can reveal the underlying adjustment mechanism during gait, which is not apparent by observing natural gait alone, and may be useful for maintaining and improving joint pain and physical function.

The primary purpose of this study was to determine the effect of the strategies used to increase gait speed on hip pain and physical function status in patients with hip OA. The secondary purpose was to examine the effects of the types of strategies used for increasing gait speed on hip loading during gait. We hypothesized that patients who increase gait speed mainly by increasing cadence would have less hip pain, a higher physical function level, and a lower rate of increase in hip joint moments.

## Methods

### Participants

Fifty-two female patients with secondary hip OA (age, 47.8 ± 10.7 years) were consecutively recruited for this study. We estimated that a minimum sample size of 42 participants was required to detect group differences (effect size, 0.5) with a power of 80% and an alpha level of 0.05, using GPower 3.1.7 (Heinrich-Heine-Universität Düsseldorf, Düsseldorf, Germany). The patients were enrolled from among patients who attended the Department of Orthopedic Surgery of a university hospital continuously from April 2013 to March 2015. The inclusion criteria were patients aged 20–65 years, who had secondary hip OA and could walk without any assistive device in daily life. The exclusion criteria were patients with a history of hip surgery (e.g., osteotomy and arthroplasty) and neurological, vascular, or other conditions that affected gait. The patient distribution among the hip OA stages [10] was pre-OA (*n* = 15, 28.8%), early-OA (*n* = 25, 48.1%), and advanced-OA stages (*n* = 12, 23.1%). Pre-OA included acetabular dysplasia, while no patient in our cohort had femoroacetabular impingement. The side with more severe radiographic OA change was used in the analysis. Written informed consent was obtained from all patients, and this study was approved by the institutional review board.

### Gait analysis

The participants wore body-fitting T-shirts and short spats. Twenty-six reflective markers were placed at various body points by a single experienced examiner. Each body segment comprised the following marker sets: the trunk, comprising the seventh cervical spinous process, the tenth thoracic spinous process, the jugular notch, the xiphoid process, and the bilateral acromioclavicular joints; the pelvis, comprising the bilateral anterior and posterior superior iliac spine; the thigh, comprising the superior aspect of the greater trochanter, and the medial and lateral femoral condyles; the shank, comprising the medial and lateral femoral condyles, and the medial and lateral malleoli; and the foot, comprising the heel, the head of the first and fifth metatarsal, and the medial and lateral malleoli. The marker position (200 Hz) and ground reaction forces (1000 Hz) were collected using an 8-camera Vicon motion system (Vicon Motion Systems Ltd., Oxford, England) and force plates (Kistler Japan Co., Ltd., Tokyo, Japan). The marker position data and ground reaction force data were filtered using a fourth-order Butterworth low-pass filter at 6 and 20 Hz, respectively. Gait speed, stride length, cadence, and external hip joint moments were computed using Vicon Nexus and BodyBuilder (Vicon Motion Systems Ltd., Oxford, England) [11]. The external hip moment peak and the hip moment impulse (timed integral of the hip joint moment) in each of the 3 planes were calculated as indexes of hip joint loading. The hip joint moment was normalized to body weight and height (Nm/kg). Stride length was expressed as a percentage of leg length (distance between the anterior superior iliac spine and medial malleolus). The mean values of the gait variables from the 3 trials were used in the analysis.

All the participants practiced normal and fast gaits several times to familiarize themselves with the experimental environment before data recording. At least 3 trials were recorded for each barefoot gait at self-selected normal (normal gait) and fast speeds (fast gait). By adjusting the start position, gait trials in which the feet properly contacted the force plates, without making the participants aware of the plate position, were secured.

**Fig. 1:**
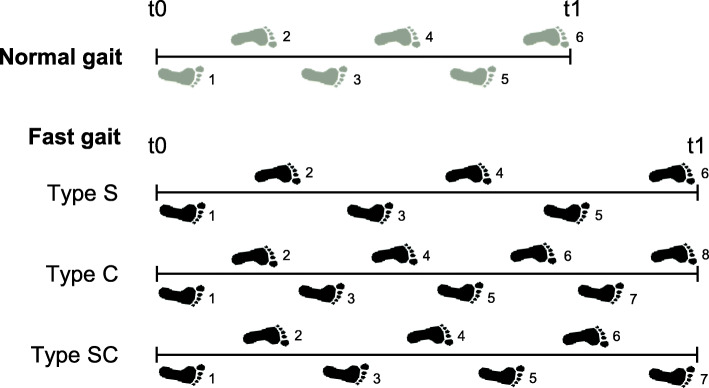
Schema of the change in each strategy type from normal to fast gaits. Type S mainly increases stride length; type C, cadence; and type SC, both stride length and cadence to increase gait speed

The types of strategies for increasing gait speed were classified according to the average stride length and cadence of 3 trials each for normal and fast gait, as described in a previous study [3]. First, the rates of increases in stride length and cadence were calculated; then, the ratio of the rate of increase in cadence to the rate of increase in stride length was computed. For the ratio, a value < 0.75 was defined as type S (i.e., increase mainly stride length), a value ≥ 1.55 as type C (i.e., increase mainly cadence), and a value ≥ 0.75 but < 1.55 as type SC (i.e., increase both stride length and cadence; Fig. 1). Participants with < 5% increase in gait speed during fast gait as compared to normal gait were excluded from the analysis.

### Assessment of hip pain and physical function

The average pain intensity at the hip joint during daily life in the last 3 months was assessed on a 100-mm visual analog scale. Physical function was assessed using the physical component summary of the Japanese version of the Medical Outcomes Study 36-Item Short-Form Health Survey (SF-36) version 2.0. SF-36 is not a disease-specific measurement tool but a generic measurement tool to assess health status; however, it has been shown to be a reliable, valid, and useful assessment tool for patients with OA [12, 13], who commonly have comorbidities [14].

### Assessment of joint space narrowing and hip impairments

A digital supine anteroposterior radiograph of the pelvis was obtained in a standardized manner by skilled radiology technicians. To assess the degree of cartilage degeneration and severity of hip OA, the minimum joint space width (mJSW) was measured digitally on the radiograph by a single examiner, using Centricity Enterprise Web, version 3.0 (GE Healthcare, Little Chalfont, England). The mJSW had the highest level of intra- and inter-rater reliabilities and good applicability as a parameter for hip OA diagnosis [15]. mJSW was measured at the vertex and medial and lateral sides of the weight-bearing surface, and if a minimum distance was present at a position other than those 3 locations, it was measured as a fourth measurement [16]. The minimum value for 3 or 4 locations was defined as the mJSW [16, 17]. The intra-rater reliability (ICC 1,1) of the mJSW measurement was 0.99 [17].

Hip range of motion (ROM) and muscle strength were assessed by a single experienced examiner, as previously reported [18, 19]. The passive ROM of the hip joint was measured at flexion, extension, and abduction, using a standard two-arm goniometer (Sakai Medical Co., Ltd, Tokyo, Japan). The intra-rater reliability (ICC 1,1) of the ROM measurements ranged from 0.82 to 0.98 [19]. Maximal isometric muscle strength on hip flexion, extension, and abduction was measured using a handheld dynamometer (μTAS F-1; Anima Co., Ltd, Tokyo, Japan). Muscle strength was measured twice, and the mean of the measurements from 2 trials was used in the analysis. The intra-rater reliability (ICC 1,1) for the muscle strength measurements ranged from 0.93 to 0.96 [19]. Muscle strength was normalized to body weight (Nm/kg).

### Statistical analyses

Differences in hip pain severity and physical function status, main outcome measures, demographic characteristics, mJSW, hip ROM, and hip muscle strength were tested using an unpaired *t*-test with Holm correction. Furthermore, as hip pain and physical function could be influenced by aging and OA severity [20–22], comparisons of these variables were also performed with adjustment for age and mJSW using a general linear model. A sensitivity analysis was also performed to evaluate the robustness of the type classification and the results of comparison of hip pain and physical function among types. Changes in gait speed, stride length, cadence, and hip joint moment were tested using the analysis of variance for split-pot factorial design (type × speed). We also calculated effect size in terms of Cohen’s *d* and *f* using GPower 3.1.7. Cohen’s *d* values of 0.20, 0.50, and 0.80 and Cohen’s *f* values of 0.10, 0.25, and 0.40 indicate small, moderate, and large effects, respectively [23]. SPSS version 26.0 (IBM Japan Ltd., Tokyo, Japan) was used for statistical analysis. The significance level was set at *P* < 0.05.

## Results

Five patients failed to achieve a gait speed change of > 5% from normal to fast gait and were excluded from further analysis; the remaining 47 patients were included. For three of the five patients, gait speed in the fast gait was slightly slower than that in normal gait. The remaining two patients were also excluded from the analysis because they failed to achieve a change in gait speed of > 5%. The results did not change significantly even when these two patients were included in the analyses (Supplementary Table 2S and Table 4S).

### Classification of the strategies for increasing gait speed

The distribution of the types of strategies was type S, 11 (23.4%); type C, 23 (48.9%); and type SC, 13 (27.7%). Four patients were of type C, with a negative increase rate in stride length (i.e., stride length decreased in fast gait compared with normal gait), despite a significantly increased gait speed. The patients’ characteristics according to strategy type are shown in Table 1. Only hip flexion ROM was significantly larger in type C than in type S.

**Table 1:** Basic characteristics in each strategy type

	Type S (*n* = 11)	Type C (*n* = 23)	Type SC (*n* = 13)
Age, years	52.3 ± 10.3	47.4 ± 11.6	46.5 ± 10.5
Height, cm	155.9 ± 5.0	158.8 ± 6.9	156.1 ± 2.5
Body weight, kg	54.9 ± 8.1	55.3 ± 8.9	52.0 ± 8.7
Body mass index, kg/m^2^	22.7 ± 3.7	22.0 ± 3.5	21.3 ± 3.3
Minimum JSW, mm	2.9 ± 1.7	3.3 ± 1.4	3.5 ± 1.2
Passive hip ROM, degrees			
Flexion	103.0 ± 15.0	118.1 ± 10.1^a^	114.0 ± 13.4
Extension	10.1 ± 3.2	12.7 ± 2.9	11.2 ± 3.3
Abduction	20.8 ± 5.7	24.8 ± 5.0	24.5 ± 6.8
Hip muscle strength, Nm/kg			
Flexion	0.77 ± 0.17	0.97 ± 0.27	0.86 ± 0.24
Extension	1.33 ± 0.21	1.69 ± 0.61	1.48 ± 0.59
Abduction	0.74 ± 0.08	0.82 ± 0.23	0.75 ± 0.21

Values are mean ± standard deviation. *JSW* joint space width, *ROM* range of motion

^a^Difference compared with type S (*P* = 0.003, effect size *d* = 1.18)

### Comparison of hip pain and physical function according to strategy type

Hip pain and physical function status according to strategy type are described in Table 2. Hip pain was not statistically significantly different between the strategy types. The physical function score was statistically significantly higher in types C and SC than in type S; this difference remained even after adjustment for age and mJSW. Statistically significant differences in physical function among types persisted in sensitivity analyses using different criteria for type classification (Table 3), indicating result robustness.

**Table 2:** Hip pain and physical function in each strategy type and comparison between strategy types

	Type S (*n* = 11)	Type C (*n* = 23)	Type SC (*n* = 13)	*P*-value* (effect size, *f*)	*P*-value* adjusted for age and mJSW (effect size, *f*)
Hip pain (VAS), mm	57.0 ± 23.5	42.8 ± 26.7	35.5 ± 26.9	S vs C, 0.284 (0.27) C vs SC, 0.436 (0.14) S vs SC, 0.150 (0.44)	S vs C, 0.502 (0.21) C vs SC, 0.524 (0.11) S vs SC, 0.312 (0.38)
Physical function (PCS in SF-36), point	28.2 ± 7.8	38.0 ± 8.8	40.6 ± 8.5	**S vs C, 0.006 (0.56)** C vs SC, 0.398 (0.15) **S vs SC, 0.003 (0.79)**	**S vs C, 0.018 (0.51)** C vs SC, 0.464 (0.13) **S vs SC, 0.015 (0.71)**

Values are mean ± standard deviation. Bold indicates statistically significant. *VAS* visual analog scale, *PCS* physical component summary

*P-value with Holm correction

**Table 3:** Sensitivity analyses of comparison between strategy types in hip pain and physical function

	Type S	Type C	Type SC	*P*-value* (effect size, *f*)	*P*-value* adjusted for age and mJSW (effect size, *f*)
**Sensitivity analysis 1**					
Type S, < 0.65 Type C, ≥ 1.45 Type SC, ≥ 0.65 and < 1.45	*N* = 10	*N* = 24	*N* = 13		
Hip pain (VAS), mm	55.9 ± 24.5	42.6 ± 26.1	37.7 ± 28.4	S vs C, 0.363 (0.24) C vs SC, 0.598 (0.09) S vs SC, 0.363 (0.35)	S vs C, 0.480 (0.22) C vs SC, 0.491 (0.12) S vs SC, 0.321 (0.39)
Physical function (PCS in SF-36), point	29.0 ± 7.7	37.7 ± 8.7	39.8 ± 9.9	**S vs C, 0.028 (0.48)** C vs SC, 0.508 (0.11) **S vs SC, 0.028 (0.62)**	**S vs C, 0.022 (0.46)** C vs SC, 0.415 (0.14) **S vs SC, 0.022 (0.69)**
**Sensitivity analysis 2**					
Type S, < 0.85 Type C, ≥ 1.65 Type SC, ≥ 0.85 and < 1.65	*N* = 11	*N* = 22	*N* = 14		
Hip pain (VAS), mm	57.0 ± 23.5	42.8 ± 27.3	36.0 ± 25.9	S vs C, 0.302 (0.26) C vs SC, 0.465 (0.13) S vs SC, 0.141 (0.44)	S vs C, 0.636 (0.19) C vs SC, 0.636 (0.16) S vs SC, 0.210 (0.42)
Physical function (PCS in SF-36), point	28.2 ± 7.8	38.6 ± 8.6	39.5 ± 9.1	**S vs C, 0.006 (0.61)** C vs SC, 0.758 (0.05) **S vs SC, 0.006 (0.69)**	**S vs C, 0.018 (0.55)** C vs SC, 0.690 (0.07) **S vs SC, 0.018 (0.66)**

The results of sensitivity analyses using the alternative criteria for type classification are shown (original criteria: type S, < 0.75; type C, ≥ 1.55; type SC, ≥ 0.75 and < 1.55). Values are mean ± standard deviation. Bold indicates statistically significant. *VAS* visual analog scale, *PCS* physical component summary

*P-value with Holm correction

### Comparison of changes in gait biomechanics according to strategy type

The changes in gait biomechanics per strategy type are shown in Table 4. No statistically significant differences among the types regarding gait speed, stride length, and cadence were found during normal gait (*P* = 0.237–0.880). Gait speed was increased in all types, and there was no main effect of type or interaction effect. Both stride length and cadence had a main effect of speed and an interaction effect. Although both stride length and cadence increased significantly in fast gait compared with normal gait in all types, the rate of increase in stride length was significantly lower for type C than for types S and SC, and that in cadence was lower for type S than for types C and SC.

**Table 4:** Changes in gait biomechanics in each strategy type and comparison between strategy types

	Type S (*n* = 11)			Type C (*n* = 23)			Type SC (*n* = 13)			*P*-value (effect size, *f*)		
	Normal	Fast	Change (%)	Normal	Fast	Change (%)	Normal	Fast	Change (%)	Type	Speed	Interaction
Gait speed, m/s	1.13 ± 0.10	1.31 ± 0.13	15.6 ± 6.9	1.19 ± 0.15	1.36 ± 0.14	15.2 ± 7.9	1.10 ± 0.16	1.31 ± 0.18	19.6 ± 7.4	0.298 (0.24)	**< 0.001 (2.34)**	0.348 (0.22)
Stride length, (% leg length)	147.2 ± 9.8	163.5 ± 17.2	10.9 ± 7.4	153.9 ± 10.1	157.4 ± 10.2	2.3 ± 2.7*	145.8 ± 13.9	158.1 ± 14.6	8.5 ± 2.8	0.651 (0.14)	**< 0.001 (1.64)**	**< 0.001 (0.89)**
Cadence, steps/min	120.4 ± 8.5	126.4 ± 12.8	4.8 ± 5.0^a^	117.0 ± 8.8	131.4 ± 10.4	12.4 ± 6.3	117.5 ± 11.2	129.4 ± 11.4	10.2 ± 4.2	0.966 (0.04)	**< 0.001 (1.72)**	**0.002 (0.57)**
Hip moment (peak), Nm/kg												
Flexion	0.47 ± 0.11	0.72 ± 0.25	53.8 ± 37.0	0.49 ± 0.11	0.68 ± 0.16	44.5 ± 32.9	0.44 ± 0.12	0.63 ± 0.22	43.5 ± 32.5	0.469 (0.19)	**< 0.001 (1.28)**	0.587 (0.16)
Extension	0.30 ± 0.10	0.35 ± 0.09	19.8 ± 13.1	0.31 ± 0.09	0.38 ± 0.08	28.0 ± 22.0	0.30 ± 0.09	0.38 ± 0.10	30.7 ± 21.5	0.889 (0.07)	**< 0.001 (1.54)**	0.177 (0.29)
Adduction	0.65 ± 0.11	0.69 ± 0.10	6.8 ± 6.7	0.70 ± 0.13	0.73 ± 0.13	4.2 ± 7.1^b^	0.65 ± 0.10	0.73 ± 0.11	13.6 ± 8.0	0.581 (0.16)	**< 0.001 (1.10)**	**0.002 (0.56)**
Internal rotation	0.09 ± 0.04	0.12 ± 0.04	34.7 ± 35.7	0.12 ± 0.04	0.14 ± 0.04	19.1 ± 19.9^b^	0.10 ± 0.04	0.14 ± 0.04	47.8 ± 34.5	0.284 (0.24)	**< 0.001 (1.47)**	**0.021 (0.44)**
External rotation	0.07 ± 0.02	0.07 ± 0.03	3.9 ± 16.5	0.07 ± 0.03	0.08 ± 0.03	7.1 ± 20.0	0.09 ± 0.02	0.10 ± 0.03	8.9 ± 26.1	0.130 (0.38)	**0.020 (0.36)**	0.786 (0.11)
Hip moment (impulse), Nm/kg												
Flexion/extension	0.09 ± 0.02	0.10 ± 0.02	3.2 ± 12.7	0.11 ± 0.03	0.11 ± 0.02	2.3 ± 9.2	0.09 ± 0.02	0.10 ± 0.02	4.1 ± 10.2	0.213 (0.27)	0.334 (0.15)	0.843 (0.09)
Abduction/adduction	0.25 ± 0.05	0.24 ± 0.05	− 7.2 ± 5.2	0.27 ± 0.05	0.24 ± 0.05	− 11.9 ± 6.9	0.26 ± 0.06	0.23 ± 0.06	− 10.9 ± 5.1	0.912 (0.06)	**< 0.001 (1.60)**	0.068 (0.36)
External/internal rotation	0.03 ± 0.01	0.03 ± 0.01	2.7 ± 12.5	0.03 ± 0.01	0.03 ± 0.01	− 2.3 ± 10.4	0.03 ± 0.01	0.03 ± 0.01	5.3 ± 14.5	0.178 (0.28)	0.345 (0.14)	0.184 (0.28)
Total	0.38 ± 0.06	0.36 ± 0.06	− 4.1 ± 4.4	0.41 ± 0.06	0.37 ± 0.06	− 7.5 ± 5.6	0.38 ± 0.06	0.36 ± 0.07	− 6.2 ± 4.2	0.561 (0.16)	**< 0.001 (1.09)**	0.139 (0.31)

Values are mean ± standard deviation. Bold indicates statistically significant

*Difference compared with type S (*P* < 0.001, effect size *d* = 1.54) and type SC (*P* < 0.001, effect size *d* = 2.25)

^a^Difference compared with type C (*P* = 0.003, effect size *d* = 1.34) and type SC (*P* = 0.018, effect size *d* = 1.17)

^b^Difference compared with type SC (adduction, *P* = 0.003, effect size *d* = 1.24; internal rotation, *P* = 0.009, effect size *d* = 1.02)

**Fig. 2:**
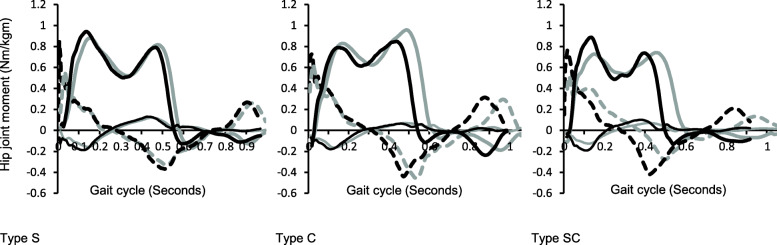
Typical waveforms of hip joint moments in types S, C, and SC. Dashed lines, thick solid lines, and thin solid lines indicate external hip flexion/extension, adduction/abduction, and external/internal rotation moments during gait, respectively. Gray colors of each represent normal gait, and black colors represent fast gait. In types C and SC, cadence is increased (i.e., stance phase time is decreased) in fast gait, and waveforms are specifically compressed on the left side in fast gait than in the normal gait

A typical example of the waveform of the hip joint moment during normal and fast gait in each strategy type is shown in Fig. 2. The peak values increased during fast gait compared with normal gait for all hip moments (Table 4). Moreover, interaction effects were found in the external hip adduction and internal rotation moments, and the increase rate was significantly lower in type C than in type SC. Regarding the hip moment impulse, although no interaction effect was observed, a speed main effect was found in the frontal plane and total hip moment impulse. The hip moment impulses were significantly decreased in the fast gait compared to the normal gait.

## Discussion

In this study, we investigated the effects of various strategies for increasing gait speed on hip pain and physical function in patients with hip OA and examined the effects of strategies for increasing gait speed on changes in hip loading during gait. Most patients with hip OA were classified as type C. Physical function status was higher in types C and SC than in type S, which suggested that the strategies that included increasing cadence to achieve faster gait speed were associated with higher physical function scores. Type C had a lower increase in peak hip joint moments, particularly hip adduction and internal rotation moments, than SC when gait speed increased. These findings partially supported our hypothesis; however, no significant difference in hip pain was found between groups based on the strategy used for increasing gait speed. To our knowledge, no previous studies have reported strategies for making changes in gait speed or their association with physical function status in patients with hip OA.

Among patients with hip OA, the highest proportion (48.9%) prioritized increasing cadence (i.e., type C) to achieve faster gait speed. This was different from the results for healthy individuals, where most subjects were of type SC (40.3%), followed by type C (32.7%) [3]. The gait of patients with hip OA is characterized by a decrease in self-selected gait speed, mainly due to a decrease in step length on the affected side and in stride length [8]. Kinematically, decreased hip flexion/extension angle is a gait feature that may decrease step and stride lengths [24]. Moreover, passive hip ROM was reportedly responsible for the variance in hip angle during gait in patients with mild-to-severe hip OA [19, 25]. These findings may explain why patients with hip OA tended to prefer to utilize the type C strategy. However, interestingly, the hip ROM in type C individuals was larger than that in type S individuals. These results indicate that the choice of cadence-increasing priority strategy observed in type C was not a compensation for peripheral constraints such as ROM restriction, but was rather a more active choice of strategy with some advantage to the stride length-increasing strategy.

In type C, the rates of increase in peak hip adduction and internal rotation moments tended to be lower than those in the other 2 types, particularly type SC. In 4 type C individuals, whose stride length decreased during fast gait, little change (0.8% increase) in peak hip adduction moment was observed despite an increase in gait speed of 11.0%. Increases in peak joint moments with increasing gait speed are known to be due mainly to the increase in stride length [3, 4, 6, 7]. In healthy individuals who used a cadence-increasing strategy, little change in hip moments was observed, except for parts of the hip flexion and adduction moments in transition from normal to fast gait [3]. The results of our study in patients with hip OA are consistent with these findings in healthy individuals. Even with the same gait speed, the anterior and posterior ground reaction forces increased when the stride increased and cadence decreased [26]. At this time, the magnitude of the vertical ground reaction force showed little change; however, the anterior and posterior inclinations of the ground reaction forces increased. Thus, changes in the magnitude and inclination of the anterior and posterior ground reaction forces cause an increase in joint moments [4, 26]. Therefore, preferential use of strategies that include increased stride length to increase gait speed tends to increase hip joint loading.

Moreover, although no interaction was observed, the hip joint moment impulse (adduction and total) decreased during fast gait in all strategy types. The hip adduction moment impulse showed the smallest increase with increasing gait speed, despite the magnitude being the largest in all 3 dimensions. Therefore, as a result of being more affected by the decreased stance time due to increased cadence, the hip adduction and total moment impulse may have decreased during fast gait. Recently, in knee OA, a lower cadence during gait (i.e., a longer stance time) has been reported to be associated with worsening of cartilage damage of the tibiofemoral and patellofemoral joints [27]. As the hip adduction moment impulse is a significant factor in generating the cumulative hip moments associated with the progression of hip OA [17], converting normal gait into fast gait over short distances might, in some cases, be useful for protecting the hip joint from degeneration.

Importantly, the physical function status was higher in types C and SC (i.e., types that involved increasing cadence) than in type S. The robustness of the results was confirmed in the sensitivity analysis, with alternative criteria of type classification. Given that increasing stride length contributes more to increasing joint load than increasing cadence, the priority of increasing stride length would increase mechanical stress on the hip joint and cause excessive energy consumption in the hip muscles in daily life and, consequently, may cause deterioration in physical function. However, no significant difference in physical function was found between types C and SC, despite the markedly lower rate of increase in hip joint moments in type C than in type SC. This suggests that the relationship between the changes in hip moment due to differences in strategies for increasing gait speed and physical function is not direct. Several factors, such as radiographic OA severity, hip and knee muscle strength, hip flexion ROM, stiffness, and pain, are associated with deteriorating physical function in patients with hip OA [20, 28–30]. In this study, the hip ROM tended to be larger in patients implementing type C than in those implementing type S. Thus, the difference in physical function in patients with hip OA should be explained on the basis of multiple factors. However, it is noteworthy that the differences in physical function between the strategy types remained statistically significant, with a large effect size, even after adjustment for hip ROM on additional analysis (type S vs. type C; *P* = 0.044, effect size *f* = 0.43: type S vs. type SC; *P* = 0.021, effect size *f* = 0.66). Moreover, differences in physical function remained significant after adjustment for gait speed (type S vs. type C; *P* = 0.014, effect size *f* = 0.52: type S vs. type SC; *P* = 0.002, effect size *f* = 0.90) or stride length (type S vs. type C; *P* = 0.032, effect size *f* = 0.46: type S vs. type SC; *P* = 0.003, effect size *f* = 0.83) during normal gait, indicating that the strategy for increasing gait speed is a factor that affects physical function independently of the characteristics of normal gait. Additionally, this difference remained significant when adjusted for BMI (type S vs. type C; *P* = 0.008, effect size *f* = 0.56: type S vs. type SC; *P* = 0.007, effect size *f* = 0.75). Thus, this study provides evidence for an important finding regarding gait-related risk factors for physical function deterioration in patients with hip OA. The difficulty in increasing cadence when increasing gait speed may be associated with deterioration of physical function. Conversely, no association was found between hip pain and strategy type in increasing gait speed. Another study reported that hip pain was not associated with hip angle and moments during gait in patients with hip OA [31]. Moreover, along with peripheral mechanisms, hypersensitivity of the central nervous system has been identified to be involved in OA pain [32]. Taken together, in this study, hip pain was affected by several other factors, and variations in the changes in mechanical loading on the hip joint and hip pain may not necessarily be associated with each other.

This study had several limitations. First, hip joint force was not directly measured despite the fact that excess hip joint forces and/or abnormal anatomy can increase cartilage damage [33]. However, the hip joint forces can be estimated using an indirect measure from three-dimensional gait analysis, and hip joint moments are strongly correlated to hip joint forces during gait [34]. Therefore, hip joint moments were used as an index of hip joint load in this study. Second, although both normal and fast gaits were measured at the self-selected speed by the patient, gait analysis was conducted in a laboratory setting. Thus, the results of this study may not necessarily reflect the change in gait speed and related gait biomechanics in patients in real life. Third, hip pain may have been caused by activities other than walking because the pain was not assessed during gait per se. Fourth, the wide range of patients’ ages and OA stages may be a limitation. However, because we performed age- and mJSW-adjusted analyses in the comparisons of hip pain and physical function, the results can be interpreted as indicating a relationship without confounding biases. Physical function was evaluated only with SF-36 (a generic measure), not a disease-specific measure such as WOMAC. Furthermore, patients with end-stage hip OA were excluded considering risks such as worsening of hip pain and task difficulty. Moreover, this study only included female patients because the patients who met the criteria had a sex bias (percentage of men, 6.9%), similar to other studies [35]. Thus, care should be taken when generalizing the results to patients with end-stage hip OA or male patients. Finally, the cross-sectional design of this study makes it difficult to consider the causal relationship between gait strategy and hip pain and physical function. Further cohort and intervention studies are needed to establish the effects of gait strategies involving changes in stride length and cadence on hip loading, hip pain, and physical function in patients with hip OA.

Despite these limitations, the study findings provide a new perspective for gait analysis in patients with hip OA. Examining not only normal gait but also the strategies used for increasing gait speed would be useful in understanding the underlying adjustment mechanism during gait, which is related to hip loading. Furthermore, the strategies can be evaluated from stride length and cadence, which can be easily measured clinically. Moreover, therapeutic exercise has been reported to increase cadence during gait in patients with hip OA and total hip arthroplasty [36, 37]. Therefore, evaluating and modifying the strategy to prioritize increasing cadence to change gait speed may contribute to the maintenance of a high physical function level.

## Conclusions

The findings of this study provide a new perspective for gait analysis in patients with hip OA. The proportion of patients who mainly increased gait speed by increasing cadence (type C) was highest among patients with hip OA. Type C individuals tended to suppress the increase in peak hip adduction and internal rotation moment with increasing gait speed. Moreover, type C and SC individuals, both of whom included increased cadence in their strategies, maintained higher physical function levels than type S, although their relationship was unclear. These results suggest that encouraging a cadence-increasing strategy for coping with changes in gait speed may be useful for reducing hip loading and maintaining and improving physical function in patients with hip OA.

## Supplementary Information

**Additional file 1: Table 2S**. Hip pain and physical function in each strategy type and comparison between strategy types. **Table 4S**. Changes in gait biomechanics in each strategy type and comparison between strategy types.

## Data Availability

All data analyzed in this study are included in this published article.

## Abbreviations

OA: Osteoarthritis
SF-36: Medical Outcomes Study 36-Item Short-Form Health Survey
mJSW: Minimum joint space width
ROM: Range of motion
ICC: Intraclass correlation
