# Supplementary material for: Strategies for increasing gait speed in patients with hip osteoarthritis: their clinical significance and effects on hip loading
Source: Arthritis Res Ther. 2021 Apr 28;23:129. doi: 10.1186/s13075-021-02514-x (PMC8080338; doi:10.1186/s13075-021-02514-x)
Supplement: Supplementary file 1 — Additional file 1: Table 2S. Hip pain and physical function in each strategy type and comparison between strategy types. Table 4S. Changes in gait biomechanics in each strategy type and comparison between strategy types. [file 13075_2021_2514_MOESM1_ESM.pdf]

Table 2S. Hip pain and physical function in each strategy type and comparison between strategy types

	Type S (n = 12)	Type C (n = 24)	Type SC (n = 13)	*P*-value* (effect size, *f*)	*P*-value* adjusted for age and mJSW (effect size, *f*)
Hip pain (VAS), mm	53.3 ± 25.7	42.4 ± 26.2	35.5 ± 26.9	S vs C: 0.450 (0.20)<br>C vs SC: 0.450 (0.13)<br>S vs SC: 0.312 (0.35)	S vs C: 0.559 (0.19)<br>C vs SC: 0.559 (0.10)<br>S vs SC: 0.396 (0.34)
Physical function (PCS in SF-36), point	28.8 ± 7.7	38.3 ± 8.7	40.6 ± 8.5	**S vs C: 0.005 (0.55)**<br>C vs SC: 0.447 (0.13)<br>**S vs SC: 0.004 (0.76)**	**S vs C: 0.009 (0.54)**<br>C vs SC: 0.532 (0.11)<br>**S vs SC: 0.010 (0.72)**

The results including the excluded two patients who failed to achieve in change in gait speed of > 5%. Values are mean ± standard deviation. **P*-value with Holm correction.

Bold indicates statistically significant. VAS = visual analogue scale. PCS = physical component summary.

Table 4S. Changes in gait biomechanics in each strategy type and comparison between strategy types

	Type S (n = 12)			Type C (n = 24)			Type SC (n = 13)			*P*-value (effect size, *f*)		
	Normal	Fast	Change (%)	Normal	Fast	Change (%)	Normal	Fast	Change (%)	Type	Speed	Interaction
Gait speed, m/sec.	1.14 ± 0.10	1.31 ± 0.12	14.6 ± 7.4	1.19 ± 0.14	1.35 ± 0.14	14.7 ± 8.1	1.10 ± 0.16	1.31 ± 0.18	19.6 ± 7.4	0.343 (0.22)	**< 0.001 (2.21)**	0.243 (0.25)
Stride length, (% leg length)	148.1 ± 9.8	163.4 ± 16.4	10.3 ± 7.4	153.9 ± 9.9	157.2 ± 10.1	2.2 ± 2.7 *	145.8 ± 13.9	158.1 ± 14.6	8.5 ± 2.8	0.627 (0.14)	**< 0.001 (1.56)**	**< 0.001 (0.85)**
Cadence, steps/min.	121.0 ± 8.4	126.6 ± 12.2	4.5 ± 4.9 †	117.0 ± 8.6	131.0 ± 10.3	12.1 ± 6.3	117.5 ± 11.2	129.4 ± 11.4	10.2 ± 4.2	0.987 (0.02)	**< 0.001 (1.67)**	**0.001 (0.58)**
Hip moment (peak), Nm/kgm												
Flexion	0.48 ± 0.11	0.73 ± 0.24	52.7 ± 35.5	0.48 ± 0.11	0.68 ± 0.16	44.5 ± 32.2	0.44 ± 0.12	0.63 ± 0.22	43.5 ± 32.5	0.427 (0.19)	**< 0.001 (1.30)**	0.574 (0.16)
Extension	0.31 ± 0.10	0.36 ± 0.09	19.8 ± 12.4	0.30 ± 0.09	0.38 ± 0.08	27.7 ± 21.6	0.30 ± 0.09	0.38 ± 0.10	30.7 ± 21.5	0.963 (0.04)	**< 0.001 (1.56)**	0.193 (0.27)
Adduction	0.65 ± 0.10	0.69 ± 0.10	6.8 ± 6.4	0.70 ± 0.13	0.72 ± 0.12	4.2 ± 6.9 ‡	0.65 ± 0.10	0.73 ± 0.11	13.6 ± 8.0	0.534 (0.17)	**< 0.001 (1.13)**	**0.002 (0.57)**
Internal rotation	0.09 ± 0.04	0.12 ± 0.04	34.3 ± 34.1	0.12 ± 0.04	0.14 ± 0.04	19.1 ± 19.4 ‡	0.10 ± 0.04	0.14 ± 0.04	47.8 ± 34.5	0.314 (0.23)	**< 0.001 (1.51)**	**0.017 (0.44)**
External rotation	0.06 ± 0.02	0.07 ± 0.03	3.9 ± 15.7	0.07 ± 0.03	0.08 ± 0.03	7.1 ± 19.5	0.09 ± 0.02	0.10 ± 0.03	8.9 ± 26.1	0.018 (0.44)	**0.017 (0.36)**	0.758 (0.11)

Hip moment (impulse), Nms/kgm												
Flexion/Extension	0.10 ± 0.03	0.10 ± 0.02	2.2 ± 12.5	0.10 ± 0.03	0.11 ± 0.02	1.8 ± 9.3	0.09 ± 0.02	0.10 ± 0.02	4.1 ± 10.2	0.367<br>(0.21)	0.288<br>(0.16)	0.838<br>(0.09)
Abduction/Adduction	0.25 ± 0.05	0.23 ± 0.05	−6.3 ± 5.7	0.27 ± 0.05	0.24 ± 0.05	−11.3 ± 7.3	0.26 ± 0.06	0.23 ± 0.06	−10.9 ± 5.1	0.804<br>(0.10)	**< 0.001**<br>**(1.63)**	0.058<br>(0.37)
External/Internal rotation	0.03 ± 0.01	0.03 ± 0.01	2.5 ± 11.9	0.03 ± 0.01	0.03 ± 0.01	−2.3 ± 10.2	0.03 ± 0.01	0.03 ± 0.01	5.3 ± 14.5	0.133<br>(0.30)	0.326<br>(0.15)	0.168<br>(0.28)
Total	0.37 ± 0.06	0.36 ± 0.06	−4.2 ± 4.2	0.40 ± 0.06	0.37 ± 0.05	−7.5 ± 5.5	0.38 ± 0.06	0.36 ± 0.07	−6.2 ± 4.2	0.547<br>(0.16)	**< 0.001**<br>**(1.12)**	0.118<br>(0.31)

The results including the excluded two patients who failed to achieve in change in gait speed of > 5%. Values are mean ± standard deviation. Bold indicates statistically significant. *: Difference compared with type S ($P < 0.001$, effect size $d = 1.45$) and type SC ($P < 0.001$, effect size $d = 2.29$). †: Difference compared with type C ($P = 0.003$, effect size $d = 1.35$) and type SC ($P = 0.018$, effect size $d = 1.25$). ‡: Difference compared with type SC (adduction, $P = 0.003$, effect size $d = 1.26$; internal rotation, $P = 0.009$, effect size $d = 1.03$).
